
JOURNAL INFORMATION
==============================
NLM Title Abbreviation: ScientificWorldJournal
Journal ID: ScientificWorldJournal
NLM Journal ID: 101131163
Journal ID: TSWJ

The Scientific World Journal

ISSN: 2356-6140
EISSN: 1537-744X
Publisher: Wiley

ARTICLE INFORMATION
==============================
PMCID: PMC6009370
PMID: 29973834
DOI: 10.1100/tsw.2002.59
Article ID: 627865
Article version: 1
Subjects: Short Report

Procollagen-Like Protein as a Molecular Target in the Treatment of Primary Brain Tumor

Wangpaichitr Medhi *
Landy Howard
Wu Chun Jing
Feun Lynn G.
Xu Rong
Xu Jenny
Savaraj Niramol
VA Medical Center and Sylvester Comprehensive Cancer Center, Miami FL 33125, USA
*Medhi Wangpaichitr: medhiw@yahoo.com
Publication date: 2002
Electronic publication date: 2002 Apr 9
Volume: 2
First page: 125
Last page: 126
Copyright: Copyright © 2002 Medhi Wangpaichitr et al.
Copyright year: 2002
License: This is an open access article distributed under the Creative Commons Attribution License, which permits unrestricted use, distribution, and reproduction in any medium, provided the original work is properly cited.
License URL: https://creativecommons.org/licenses/by/3.0/

==============================
Advances in Gene Technology: The Genome and Beyond –
Structural Biology for Medicine (Proceedings of the 2002 Miami
*Nature Biotechnology* Winter Symposium)
*TheScientificWorld* 2002, 2(S2), 125–126
ISSN 1532-2246; DOI 10.1100/tsw.2002.59

### PROCOLLAGEN-LIKE PROTEIN AS A MOLECULAR TARGET IN THE TREATMENT OF PRIMARY BRAIN TUMOR

Medhi Wangpaichitr, Howard Landy, Chun Jing Wu, Lynn G. Feun, Rong Xu, Jenny Xu, and Niramol Savaraj
VA Medical Center and Sylvester Comprehensive Cancer Center, Miami FL 33125
medhiw@yahoo.com

**INTRODUCTION.** We have previously reported that the presence of a unique 220-Kd protein, which is recognized by vitamin D receptor (VDR) monoclonal antibody, correlated with vitamin D3 (VD3) sensitivity in primary brain tumor[1]. In this communication we have attempted to further characterize this protein and to investigate how VD3 induces apoptosis in glioma cells.

**METHODS.** cDNA library was obtained in one glioma cell line which highly expressed this protein and screening was performed using VDR monoclonal antibody. This partial cDNA was then subcloned into pcDNA3.1 and sequencing was performed. This partial cDNA sequence also was used as a probe to identify mRNA, which encodes for this putative gene. To characterize this 220-Kd protein, we have used five cell lines as follows: Glioma 1, U118, SW1783, which possess variable amount of 220-Kd protein, and U-373 and T-98G which lack this 220-Kd protein.

To study the mechanism how VD3 induces apoptosis, we have employed the gene array technique to identify which apoptotic pathway(s) may be involved in this event

**RESULTS.** We have identified a 2.2-Kb fragment using immunoscreening. This fragment was subsequently sequenced. Via BLAST search through GENBANK, this cDNA fragment was found to be identical to nucleotide 2840-5118 of procollagen alpha 1 type 1. These nucleotides correspond to the C-terminal of procollagen and 600 bp of 3'untranslated region. It is unclear why VDR monoclonal antibody recognizes the C-terminal of procollagen. To clarify this, we have used the immunoprecipitation technique to investigate whether a VDR monoclonal antibody (obtained from Chemicon) cross reacts with procollagen type 1. Our results indicate that the immunoprecipitant of cell lysate with VDR antibody also reacts with the antibody against the C-terminal peptide of procollagen type 1. Furthermore, immunoblot of cell lysate using antibody against N- and C-terminal of procollagen type 1 also detected the 220-Kd protein. The signal intensity obtained from the five cell lines also correlates with those obtained using VDR monoclonal antibody.

Using the 1.6 Kb as a probe, we have performed northernblot analysis on these five cell lines. Our results are shown below.

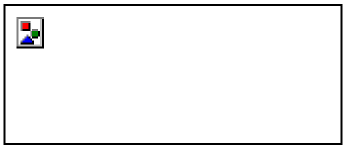

1 2 3 4 5

Lane 1: SW1783; Lane 2: Glioma; Lane 3: U118; Lane 4: T98G; Lane 5: U373

This 1.6-Kb fragment hybridized to 5.8-Kb message, which is similar to those reported in the literature for procollagen type 1.

To study how VD3 induced apoptosis in three cell lines, which possess this 220-Kd protein, cDNA was obtained from control as well as from cells after exposure to 10 ng of VD3 for 48 h. This cDNA was labeled with $^{32}$dCTP and hybridized with membrane, which contained genes from different signal transduction pathways and apoptosis pathways. These membranes were obtained from SuperArray. Our results indicated that apoptosis might involve caspase-9 but not 3. Inhibitor for caspase 3 also did not inhibit apoptosis.

**DISCUSSION.** We have previously identified a 220-Kd correlates with VD3 sensitivity. In this report, we have shown that this protein is also recognized by procollagen antibody to both N- and C-terminal. However, whether this protein is indeed procollagen type 1 or not is still unclear. It has been reported that procollagen type 1 contains two mRNA species at 4.8 and 5.8 Kb, respectively[2,3], thus it is conceivable that the 5.8 Kb mRNA could be procollagen type 1. The molecular weight of procollagen alpha 1 type 1 is unclear. An extensive review of the current literature suggests that the molecular weight of this protein varies from 140–210 Kd. These differences may represent different mRNA species found in different types of tissues as well as post-translational modification. However, our initial results do not suggest that the 220-Kd protein is glycosylated or phosphorylated. Preliminary data suggest that the putative full length cDNA also possesses sequence which is homologous to the N-terminal of procollagen alpha 1 type 1, but differences are seen starting from nucleotide 1777-2840. These data need to be confirmed. Nevertheless, the expression of this 220-Kd protein detected by either RT-PCR or immunoblot using procollagen type 1 antibody or VDR monoclonal antibody strongly correlated with *in vitro* vitamin D sensitivity. Further work is ongoing in our laboratory to isolate the full-length cDNA and to study its relationship with VD3 sensitivity.

**REFERENCES**

1. Zou, J., Landy, H., Feun, L., Xu, R., Limpidis, T., Wu, C.J., Furst, A.J., and Savaraj, N. (2000) J. Biochem. Pharmacol. 60, 1361–1365.
2. Chu, M.L., Wet, W., Bernard, M., and Ramirez, F. (1985) J. Biol. Chem. 260(4), 2315–2320.
3. Tromp, G., Kuivaniemi, H., Stacey, A., Shikata, H., Baldwin, C.T., Jaenisch, R., and Prockop, D.J. (1988) Biochem. J. 253(3), 919–922.
